# Integration of maternal postpartum services in maternal and child health services in Kaya health district (Burkina Faso): an intervention time trend analysis

**DOI:** 10.1186/s12913-018-3098-6

**Published:** 2018-04-23

**Authors:** Danielle Yugbaré Belemsaga, Anne Goujon, Halima Tougri, Abou Coulibaly, Olivier Degomme, Els Duysburgh, Marleen Temmerman, Seni Kouanda

**Affiliations:** 1 Biomedical and Public Health Department, Institut de Recherche en Sciences de la Santé, 03 B.P 7192, Ouagadougou 03, Burkina Faso; 2 Wittgenstein Centre for Demography and Global Human Capital (IIASA, VID/OAW, WU), Vienna, Austria; 3 International Centre for Reproductive Health, Faculty of Medicine and Health Sciences, Department of Uro-Gynaecology, Ghent University, Ghent, Belgium; 4 Centre of Excellence in Women and Child Health, Aga Khan University, Nairobi, Kenya; 5 African Institute of Public Health, Ouagadougou, Burkina Faso

**Keywords:** Postpartum, Postnatal, Maternal and infant health, Integration of services, Burkina Faso

## Abstract

**Background:**

The *Missed Opportunities in Maternal and Infant Health (MOMI)* project aimed at reducing maternal and newborn mortality and morbidity within the year after childbirth in four sub-Saharan African countries. MOMI interventions including the integration of maternal and infant services in the postpartum (PP) period at day 6–10, week 6–8, and month 9 were implemented from September 2013 to December 2015. We hereby assess the effect of integrating maternal postpartum care (PPC) in infant immunization services in Kaya health district in Burkina Faso.

**Methods:**

We apply a longitudinal mixed method on monthly monitoring data collected from 12 months before the project start to the end. Outcome indicators were: attendance of PPC at day 6–10 and week 6–8, provision of PP family planning counselling, and management of PP morbidity in mother and infant. We tested the significance level of changes in the different indicators by performing an interrupted time series analysis with Newey–West standard errors and one lag. Additional data were extracted at the individual level which allowed to link infant immunization with maternal PPC from September 2013 to August 2014 in the health facilities’ (HF) PP and immunization registers. We also conducted a review of documents that allowed for a qualitative evaluation of the effects.

**Results:**

Show an increasing trend of all monitored indicators during the interventions, particularly at day 6–10 when PPC increased from 61% in 2013 to 81% in 2015 and especially in rural areas (*p* < 0.05). We found large improvements in the detection and management of PP maternal hemorrhage, sepsis and newborn fever or low temperature. However, the intervention was less successful in raising PPC at week 6–8 and later due to the existence of structural barriers, caused for instance by the lack of collaboration among health workers and high turnover in the staff of HF.

**Conclusion:**

The overall package of community and facility interventions contributed to improve integrated PPC at day 6–10, particularly the role of community health workers. While the integration of maternal and child health services seems to be a valid concept, it needs to be rethought within the primary health care system.

## Background

The sustainable development goal (SDG) number 3 about health and well-being specifically addresses reproductive, maternal, newborn and child health (RMNCH) issues under Targets 3.1, 3.2, and 3.7. Together with the findings from previous studies—that postpartum care (PPC) was one of the neglected components of RMNCH services which require improvement [1–3]—emerged the idea of scaling up integrated packages of essential services across the continuum of care delivered through three platforms: communities, primary health care facilities and hospitals. There is evidence of successful experiences of integration for instance in the field of family planning (FP) on the one hand with human immunodeficiency virus/acquired immunodeficiency syndrome (HIV/AIDS) treatment and on the other with immunization services for instance in the case of the Philippines, Rwanda and Togo [4–6]. Undeniably, integrating services was shown to be a valid and cost effective intervention [7, 8].

Within the Missed Opportunities in Maternal and Infant Health (MOMI) project, a context-specific intervention aimed at integrating maternal postpartum services in child immunization services was implemented. MOMI’s overall objective was to reduce maternal and newborn mortality and morbidity within the year after childbirth, through the following three tailored interventions [2, 5, 9]: (i) Female community health workers (CHWs) who were traditionally accompanying women up to delivery would be additionally supporting mothers and infants during the PP period. In this framework, female CHWs were conducting home visits, providing individual counselling and group sensitization on danger signs in mothers and infants), referring mothers and infants for postpartum visits and when dangers signs were found, and providing counselling on FP; (ii) The delivery of immediate PPC in health facilities was enhanced with focus on the detection and management of postpartum haemorrhage and sepsis; (iii) Maternal and infant PPC (including FP counselling and provision) were integrated to child vaccination in health facilities.

Those measures were intended to strengthen the health system with notable cross-cutting benefits beyond PPC encompassing a broad range of RMNCH issues [9–11]. In addressing policy and organizational constraints, it proposed to strengthen relationships between the building blocks composing the health system. This required a comprehensive set of combined interventions that complemented and reinforced one another, particularly in maternal health as demonstrated in Rwanda for instance [12].

The main rationale behind this intervention is that while women tend to follow the vaccination schedule for their infant, they are less likely to have themselves checked in the PP period. The implementation plan specifies that each opportunity should be used to provide PPC to the mother–infant pair following the protocols, which meant that at the time of infant immunization, the infant health book was checked to see whether the mother had received PPC as well already, otherwise she would either receive PPC or be directed to it.

In this paper, we evaluate the effect of integrating maternal PPC in infant immunization services through the analysis of the monitoring qualitative and quantitative data.

## Method

### Context

The study is based on data collected in the MOMI project sites in the Kaya health district, which is located in the Centre Nord health region of Burkina Faso. The Kaya health district had 564,867 inhabitants and 52 primary HFs in 2014 [13]. The MOMI project was implemented in 12 HFs including the Kaya Demographic and Health Surveillance System (Kaya HDSS) areas.

### Description of the intervention

The overall objective of the package of three tailored interventions was to improve maternal and newborn health through a focus on the postpartum (PP) period, adopting context-specific strategies to strengthen health care provision and services at both facility and community level. The intervention focused on 12 health facilities (HFs) and 69 surrounding communities during the period from October 2013 to December 2015 [2, 14]. The specific objectives were: (i) to increase PP visit rates from 19% in 2011 to 50% in 2015 within the first 7 days after birth; (ii) from 19% in 2009 to 50% in 2015 at week 6–8 after birth; and (iii) to increase PP family planning (FP) utilization rate from 21% in 2011 to 40% in 2015 [14].

The PPC checkup focused on danger signs in mothers (postpartum hemorrhage (PPH), sepsis, anaemia), FP and dangers signs in infants, along the following schedule:

- for mothers: prevention, management and treatment of PPH at day 6–10*;* detection and management of postpartum sepsis at day 6–10*;* detection, management and treatment of postpartum anaemia at day 6–10*,* week 6–8*,* and month 9*;* and advice on (and provision of) postpartum FP at week 6–8 and month 9*.*
- for infants: detection management and treatment of infant danger signs at day 6–10*,* week 6–8*,* and month 9*.*

PPC for the mother–infant pair was integrated at three occasions with infant immunization: (i) PPC at day 6–10 integrated with the administration of bacille Calmette-Guérin (BCG) and polio zero dose vaccines; (ii) PPC at week 6–8 integrated with giving diphtheria, tetanus, pertussis in combination with inactivated polio vaccines, haemophilus influenza type b, rotavirus and pneumococcal conjugate/dose 1, and (iii) PPC at month 9 integrated with vaccinating against infant measles and yellow fever.

The objectively verifiable indicators were the number of mother–infant dyads who received PPC at day 6–10, at week 6–8 and/or at month 9–12 and the number of women who chose an FP method during PPFP counselling^1^ [14].

The implementation of the MOMI package of activities followed several steps including specific activities for the integration of PPC within child clinics:

- Preparatory activities and meetings with HF managers and female community health workers (CHWs), and personnel in charge of immunization and maternal health; workshops with health workers in each facility (July 2013).
- Training and retraining of facility health workers (FHWs) on PPC interventions within the MOMI project including integration (September 2013, December 2013 and March 2015); FHWs started the implementation of the intervention in September 2013.
- Development and distribution of PPC checklists for FHWs, and training in their use (20 January to 5 February 2014); the checklists detailed the contents of the visit for mother and infant.
- Quarterly supervision from October 2013 to December 2015 covering the provision of activities according to checklists, monitoring of the outputs, discussion and solving problems of HWs in order to maximize their performance [15].

During the implementation non-financial incentives were distributed to HF staff, e.g. blouses (May 2014) and certificates of participation in the MOMI project (January 2016).

### Study design

We conducted a longitudinal case study using mixed method before and after the intervention implementation [16]. A log frame with a schedule of activities, e.g. training sessions, development of checklists, supervision & monitoring plan, was set up before the intervention implementation [14, 17].

### Data collection

The monitored indicators cover the period from one year before the intervention implementation (baseline in September 2012) to three years until the end of the project, in December 2015. The context-specific activities were designed and implemented as to continue after the research project. Data were extracted from the HFs’ records such as PP and immunization registers, and from documents provided by the research team, e.g. supervision reports, event logs and others.

Monthly data were collected by the research team on a quarterly basis. Some additional data were extracted at individual level with dates for link between infant immunization and mother PPC from September 2013 to August 2014 in the HFs’ PP and immunization registers. Since PP visit at month 9–12 and the integrated care with infant immunization was a challenge because of reporting issues, we focused the trend analysis on the two milestones of day 6–10 and week 6–8. Data by source and content are presented in Additional file 1.

### Data analysis

We realized a descriptive univariate and bivariate analysis of the study indicators as ratio to the total number of live births. The indicators were: ratio of women who utilized day 6–10 PPC, ratio of women who utilized week 6–8 PPC, ratio of women who chose any PPFP method during the counselling.

Then we performed an interrupted time series analysis (ITSA) to assess the effect of the intervention in increasing PPC delivery at day 6–10 and week 6–8, in the choice of PPFP methods, using a single-group design in rural (Basnere, Damesma, Delga, Namsigui, Napalgue, Lebda, Kalambaogo, Tangasgo) and urban areas (Sector 1, Sector 4, Sector 6, Sector 7) of the intervention sites [18]. More specifically, we assessed whether the implementation of a package of interventions by MOMI resulted in a shift in the level and trend of the related indicators compared with those of the pre-intervention period. We analysed the effect immediately after the intervention and the effect on the slope (trend t) which is the effect on the future evolution of the dependent variable. In the estimation of the model we assumed that there is a serial autocorrelation of order 1. To ensure the validity of this hypothesis we use the *actest* of Baum and Shaffer [19]. Since there is a seasonality issue with the numbers of live birth deliveries used for the denominator, we adjusted the model to account for autocorrelation (lag of 12 months). Indeed, women become pregnant during the dry season, mainly from December to January, during which farming activities are less intense and temperatures are the lowest. Therefore, live birth deliveries were more numerous in September 2013, 2014 and 2015 (Additional file 2).

The regression analysis was done in the following steps: first, we specified a single-group ITSA with each indicator per area (rural, urban) as treatment group, and the start of the intervention on period 13 (September 2013); then we requested post-intervention trend estimates and plotted the results for the analysis. The model is estimated using Newey regression with a 12-month lag. To estimate this regression model form^2^ we used the Stata routine developed by Linden (2015) [18]. The error in the formula allows the econometric model to capture all factors. The method suits our study as it shows the trend break, the effect immediately after the intervention and the effect in terms of change in trends.

The methods above mentioned aim at analysing PPC utilization for the mother–infant pair but do not inform about the particular integration of maternal PPC in child immunization services. Therefore, and with the aim to measure the level of integration, we linked maternal PPC registers and immunization registers by creating a label using information on HFs, mothers’ names and their infants’ dates of birth. Based on this label, the linkage was established using the VLOOKUP function in Microsoft Office Excel to match PPC visits with immunization visits at the several identified milestones, e.g. Day 6–10 PPC and BCG vaccine dates.

Table 1 presents the results of the data linkage of infant immunization and PP services as presented in both registers, meaning the women who benefitted from PPC and whose infant received the recommended immunization. Thirty-seven percent (*n* = 4671) of the data could be linked for all 12 health facilities. In urban health facilities, we found the lowest percentage of matching records (only 13–29% could be linked) but with a large number of infants who were immunized, ranging from 1822 infants in Sector 1 to 541 in Sector 7. In rural health facilities except Namsigui (37%, *n* = 187), the share of the data that could be linked ranged from 50% to 72%. It is important to note that the low rate of matching records between both registers could either be interpreted as low quality in the registration system or low use of mother PPC, hence the analysis that follows should be taken with caution.

**Table 1:** Linkage of primary health facilities infant immunization and PP services registers data

Primary health facility		Infants immunization N	Linked data	
			n	%
Rural HF	Basnere	396	237	60%
	Damesma	263	150	57%
	Delga	401	289	72%
	Kalambaogo	532	271	55%
	Lebda	494	271	55%
	Namsigui	506	187	37%
	Napalgue	322	192	60%
	Tangasgo	186	117	63%
	*Total rural HF*	*3100*	*1714*	*55%*
Urban HF	Sector 1	1822	454	25%
	Sector 4	625	181	29%
	Sector 6	1357	353	26%
	Sector 7	541	72	13%
	*Total urban HF*	*4345*	*1060*	*24%*
*Total*		*7445*	*2774*	*37%*

We then performed a descriptive and bivariate analysis of the linked data using Excel and SPSS 22.

Beside the quantitative analysis above mentioned, we realized a document review of supervision reports and other reports related to the implementation of MOMI interventions and linked them to the target intervention on integration.

## Results

### Description of the sample/data

#### Monitored indicators

Table 2 present the monitoring data for the whole study period and by period for the 12 primary HFs in rural (Basnere, Damesma, Delga, Kalembaogo, Lebda, Namsigui, Napalgue, Tangasgo) and urban areas (Sector 11, Sector 4, Sector 6 and Sector 7). In the Kaya health district and for the 40 months of observation, 15,222 lives births were reported. The average utilization rate of PPC at day 6–10 was 61% (*n* = 9281) and 35% (*n* = 5421) at week 6–8.

**Table 2:** Monitored indicators and number of monthly observations by health facility for the study period (Sept 2012-Dec2015)

Primary health facilities		Number of live birth deliveries	Day 6–10 PPC				Week 6–8 PPC				PPFP choice			
		N	n	%	Monthly observations		n	%	Monthly observations		n	%	Monthly observations	
					n	%			n	%			n	%
Rural HF	Basnere	1295	1110	86%	36	90%	732	57%	36	90%	186	14%	40	100%
	Damesma	701	466	66%	36	90%	189	27%	36	90%	174	25%	24	60%
	Delga	950	672	71%	34	85%	539	57%	35	88%	133	14%	26	65%
	Kalambaogo	1278	1046	82%	37	93%	924	72%	37	93%	196	15%	40	100%
	Lebda	953	842	88%	37	93%	642	67%	37	93%	139	15%	22	55%
	Namsigui	1289	657	51%	28	70%	481	37%	28	70%	137	11%	24	60%
	Napalgue	962	620	64%	34	85%	462	48%	32	80%	128	13%	21	53%
	Tangasco	501	380	76%	35	88%	260	52%	33	83%	119	24%	21	53%
	*TotaL rural*	*7929*	*5793*	*73%*	*37*	*93%*	*4229*	*53%*	*37*	*93%*	*1212*	*15%*	*40*	*100%*
Urban HF	Sector 1	2512	784	31%	28	70%	240	10%	27	68%	393	16%	26	65%
	Sector 4	1421	1089	77%	40	100%	424	30%	40	100%	220	15%	27	68%
	Sector 6	1837	830	45%	36	90%	320	17%	32	80%	262	14%	26	65%
	Sector 7	1573	785	50%	30	75%	208	13%	30	75%	345	22%	29	73%
	*Total urban*	*7343*	*3488*	*48%*	*40*	*100%*	*1192*	*16%*	*40*	*100%*	*1220*	*17%*	*28*	*70%*
*TOTAL HF*		*15,272*	*9281*	*61%*	*40*	*100%*	*5421*	*35%*	*40*	*100%*	*2432*	*16%*	*40*	*100%*

Usually, women did not choose an FP method immediately at the time of counselling: the share of women who chose a PPFP was only 16% (2432). However the quality of the PPFP data did not allow for a meaningful analysis for several reasons i.e. lack of data before the intervention, or after the intervention particularly in urban HFs.

Monitored indicators by number of cases by health facility per period are presented in Additional file 3.

### Effects of the package of interventions on the utilization of PP services

The results present the effects of the package of interventions on the utilization of PP services by place of residence (using an ITSA) and by primary HF from September 2012 (period 1) to December 2015 (period 40), knowing that the MOMI intervention started in September 2013 (period 13).

#### Results per primary health facilities

Table 3 shows that the above findings are not homogenous across all health facilities. For day 6–10, the increase was very strong in the first year after implementation in both rural and urban facilities, except in the Lebda HF where the initial level had been already quite high (73%) and the increase was meagre (2 percentage points). For some HFs, the leap was very strong after the implementation, like in Delga rural HF from 10% to 92%, or in the urban HF of Sector 7 from 6% to 62%. In the third period (September 2014–August 2015), the increase was generally more moderate. The shares declined in the last period (September 2015–December 2015), because of a general strike that took place throughout the national territory from 17 to 28 September 2015 and also affected the health workers. In Damesma, Delga and Sector 4 the share of mother–infant pairs who visited PPC services at day 6–10 decreased from 93%, 94%, and 80% (from September 2014–August 2015) to 77%, 57%, and 68%, respectively. Nevertheless with the exception of the urban HF Sector 1, all HFs achieved the 50% target for PPC at day 6–8. The fact that the integration was particularly successful in rural areas can to a large extent be attributed to the upstream work of female CHWs in rural areas who were able to support and encourage women to visit PPC. The complexity of slum structures in the urban areas did not facilitate the work of the female CHWs. While 6 out of 8 rural HF managed to reach the 50% utilization rate target for week 6–8 PP—Damesma HF with 8% and Delga HF with 43% are the exceptions—none of the urban facilities met the target. Most of them actually fell far off the mark, with a share below 25%.

**Table 3:** Monitored indicators in proportion of live births deliveries by health facility per period

Primary health facilities		% of pair mother newborn/infant who received postpartum care at day 6–10				% of pair mother newborn/infant who received postpartum care at week 6–8				% of women who used a PPFP method			
		Sept12-Aug13	Sept13-Aug14	Sept14-Aug15	Sept15-Dec15	Sept12-Aug13	Sept13-Aug14	Sept14-Aug15	Sept15-Dec15	Sept12-Aug13	Sept13-Aug14	Sept14-Aug15	Sept15-Dec15
Rural HF	Basnere	43%	99%	98%	100%	16%	61%	78%	73%	2%	3%	29%	29%
	Damesma	16%	83%	93%	77%	5%	35%	46%	8%	0%	36%	42%	5%
	Delga	10%	92%	94%	57%	11%	63%	85%	43%	0%	13%	26%	8%
	Kalambaogo	50%	92%	90%	103%	53%	74%	81%	87%	19%	11%	19%	7%
	Lebda	72%	74%	116%	98%	59%	44%	94%	90%	0%	5%	30%	37%
	Namsigui	0%	51%	82%	84%	0%	17%	72%	78%	0%	7%	9%	49%
	Napalgue	17%	70%	91%	93%	12%	32%	86%	80%	0%	9%	27%	24%
	Tangasco	25%	96%	110%	91%	5%	54%	82%	109%	0%	8%	40%	92%
	*Total rural HF*	*30%*	*81%*	*95%*	*92%*	*21%*	*48%*	*79%*	*75%*	*3%*	*11%*	*25%*	*30%*
Urban HF	Sector 1	0%	39%	46%	49%	0%	11%	13%	21%	0%	7%	35%	23%
	Sector 4	79%	75%	80%	68%	35%	31%	26%	25%	0%	10%	34%	13%
	Sector 6	15%	43%	62%	63%	1%	11%	28%	38%	0%	11%	30%	6%
	Sector 7	6%	62%	67%	70%	1%	16%	17%	24%	0%	10%	41%	43%
	*Total urban HF*	*20%*	*51%*	*61%*	*61%*	*7%*	*16%*	*20%*	*27%*	*0%*	*9%*	*35%*	*22%*
*Total HF*		*25%*	*67%*	*78%*	*76%*	*14%*	*33%*	*50%*	*51%*	*2%*	*10%*	*30%*	*26%*

While the rate of women who chose to use a PPFP method was low, it nevertheless increased from September 2013 to August 2015 in all HFs. Indeed often the woman asked to think and to consult with her husband before deciding for a contraceptive method. Also, even if a choice was made at the time of the visit, this does not mean that the woman actually used a contraceptive method in the end.

#### Results per place of residence: ITSA

Table 4 shows the result of the regression analysis for day 6–10 PPC, week 6–8 PPC in rural and urban primary HFs sites. PPFP proposal and choice are only for rural sites since data before the intervention were missing for urban areas.

**Table 4:** Interrupted time series analysis for day 6–10, week 6–8 PPC and PPFP choice by residence

		Day 6-10 PPC		Week 6-8 PPC		PPFP choice
		Rural	Urban	Rural	Urban	Rural
		(1)	(2)	(3)	(4)	(5)
*Variables*						
T	Time since the start of study (September 2012-December 2015)	3.952***	3.388***	4.086***	1.302***	-0.113
		(0.818)	(0.181)	(0.411)	(0.0824)	(0.0886)
X	Effect immediately after the intervention initiation	17.21***	7.341**	-8.195	-3.678*	1.760
		(4.211)	(2.419)	(5.200)	(1.657)	(1.080)
X*T	Interaction of the intervention and the time	-3.114**	-2.810***	-2.261***	-0.735***	1.247***
		(0.958)	(0.194)	(0.492)	(0.123)	(0.148)
Constant		20.64***	-2.186	8.116**	-1.394*	4.031***
		(5.127)	(1.550)	(2.566)	(0.528)	(0.731)
Treated: _b[T]+_b[X*T]	*Intervention trend*	0.8381 **	0.5787***	1.8250***	0.5676***	1.1335***
		(0.2738)	(0.1100)	(0.3267)	(0.1038)	(0.1099)
*Number of monthly observations*		37	40	37	40	40

Note: Standard errors in parenthesis; *p*-value: *p<0.05, **p<0.01, ***p<0.001

**Fig. 1:**
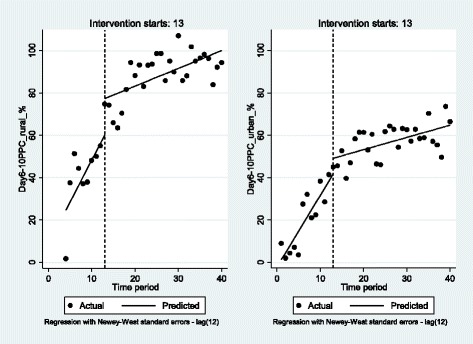
Interrupted time series analysis for day 6-10 PPC in rural and urban primary HFs Note: Some shares are above 100% due to discrepancies between the place of delivery and the PPC visit

The day 6–10 PP checkup in rural and in urban areas increased significantly by 4.0 (*p* < 0.05) and 3.4 (*p* < 0.001) percentage points per month, respectively. In the first month of the intervention, the package of interventions contributed significantly to increasing the utilization rate of PPC by mothers and infants by 17.2 (*p* < 0.05) and 7.3 percentage points. However the rate of improvement (as shown by the linear trend lines on Fig. 1) was not as important as before the intervention both in rural and urban areas by 3.1 and 2.8 (*p* < 0.001) percentage points to lie at 0.8 (*p* < 0.01) and 0.6 (*p* < 0.01) percentage points. Indeed, the increase in the proportion of mother–infant pairs who received PPC had already started before the intervention as a result of steady improvements in routine activities. Between September 2013 and December 2015, the intervention was able to continue the increase but at a slower pace compared to the past in both rural and urban primary HFs. Since the end of 2014, the day 6–10 PPC utilization rate has been close to 100% in rural HFs. It seems to stagnate around 60% in urban HFs. The decline in the monthly trend of the day 6–10 PP checkup can be explained by a saturation effect. Indeed, in the first phase of implementation, MOMI managed to raise awareness and convince women who would not have participated before for lack of information. However, the closer the proportion of women participating in the program was to 100%, the harder it was to attract the remaining group of mothers, who might be aware of the program and its benefits but did not participate for social or cultural reasons. Figure 1 illustrates these trends.

**Fig. 2:**
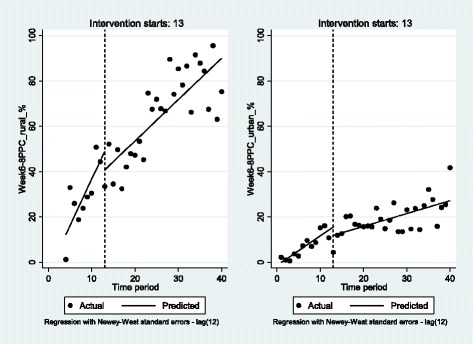
Interrupted time series analysis for week 6-8 PPC in rural and urban primary HFs

The week 6–8 PPC utilization rate in rural and urban sites was upgraded significantly by 4.1 (*p* < 0.001) and 1.3 (*p* < 0.001) percentage points per month. The increase was less after the intervention start: to 1.8 (*p* < 0.001) and 0.6 (*p* < 0.01) percentage points. While the target of 50% was achieved overall and in rural primary HFs sites, urban areas did not reach the objective by December 2015 although the increase is clearly visible there as well (Fig. 2).

**Fig. 3:**
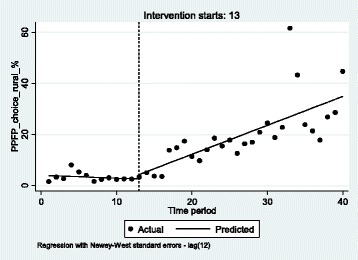
Interrupted time series analysis for PPFP choice in rural primary HFs

Figure 3 shows that information and counselling about PPFP methods did not automatically translate into actions as the share of women choosing an FP method during the PP period did not exceed 36% by the end of December 2015. This result was nevertheless close to the objective of 40% FP utilization rate by 2015. The noticeable peaks in the data are related to specific activities, for instance the National FP Week (from 18 to 23 May 2015) in which FP supplies was free of charge for women and NGOs’ activities in primary health facilities such as those coordinated by Marie Stopes International (from June to September 2015).

We performed a sensitivity analysis using the numerators of the indicator and the log (100-indicator in %). We find the same results in the trend.

### Integration of mother PP services into infant immunization services

Table 5 shows the utilization rate of day 6–10 PP visits and its integration with BCG vaccines in the different HFs. Day 6–10 PP visit for linked data rates ranged from 80% in Kalambaogo to 95% in Damesma. BCG vaccines and day 6–10 PP visit were carried out on the same day only for 10% (*n* = 272) of all HFs. The highest integration rate was in rural Napalgue (22%, *n* = 42) and in urban Sector 1 (16%, *n* = 72).

**Table 5:** Day 6–-10 PP visit and its Integration with BCG vaccines in 12 primary health facilities

Primary health facility		Linked data	Day 6–10 PP visit (Yes)			Integrated BCG and day 6–10 PP visit (Yes)		
		N	n	%	*p*	n	%	*p*
Rural HF	Basnere	237	209	88%	***	27	11%	***
	Damesma	150	143	95%		19	13%	
	Delga	289	243	84%		15	5%	
	Kalambaogo	271	217	80%		2	1%	
	Lebda	271	258	95%		21	8%	
	Namsigui	187	160	86%		27	14%	
	Napalgue	192	175	91%		42	22%	
	Tangasgo	117	100	85%		4	3%	
	*Total rural HF*	*1714*	*1505*	*88%*		*157*	*9%*	
Urban HF	Sector 1	454	404	89%		72	16%	
	Sector 4	181	151	83%		0	0%	
	Sector 6	353	291	82%		41	12%	
	Sector 7	72	64	89%		2	3%	
	*Total urban HF*	*1060*	*910*	*86%*		*115*	*11%*	
*Total*		*2774*	*2415*	*87%*		*272*	*10%*	

Note: *p*-value: ***p<0.001

The week 6–8 PP visit for linked data is 29% (805/2774) for all 12 HFs. It is higher in rural HFs and more than 50% in Basnere, Delga and Lebda. The numbers were very low and we could not find any evidence of integration with infant immunization.

Comparison of data from the two methods used were provided in Additional file 4.

### Effect of the intervention on the management of PP morbidity in mothers and their child

The MOMI project interventions aimed to enhance the management of postpartum morbidity in mothers (PPH, sepsis and anaemia) and in their children (newborn fever, low temperature and prematurity).

**Fig. 4:**
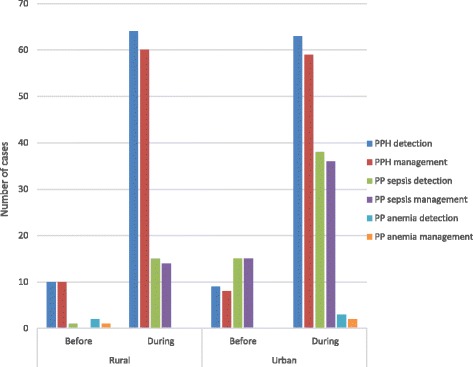
Detection and management of PP haemorrhage, sepsis and anaemia

Figure 4 shows the number of cases of PPH, sepsis and anaemia that were detected and managed before (from September 2012 to August 2013) and after the intervention (from September 2013 to December 2015), showing considerable improvements with the intervention as well as similarities between rural and urban: the health facilities registered an increase in the number of outpatient visits particularly for primary PPH but also for sepsis. The low number of detected anaemia cases could be related to the detection method used (inspecting conjunctive and palmar pallor) which could have led to underdiagnoses. Almost all detected cases were managed following the protocol.

**Fig. 5:**
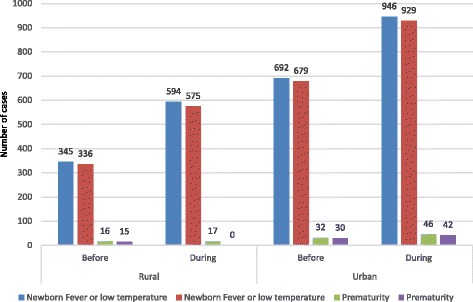
Detection and management of newborn fever or low temperature and prematurity

Figure 5 presents the detection and management of newborn fever or low temperature and prematurity. There was tremendous improvement for the first two danger signs, but less so for prematurity. Management almost universally followed detection.

The reported numbers have to be taken with utmost care as they are quite low and possibly do not include all morbidity cases. The problem could be a sign that HFs were not utilized to their full capacity. On the other hand, it could also be linked to problems of completeness in the HF retrospective survey.

### Qualitative analysis result of the intervention outcome

The review of the MOMI project documents such as supervision reports allowed for the qualitative analysis of the intervention implementation. During the intervention several issues related to the implementation of activities were gradually improved. This was done mainly during the training and recycling sessions of health workers on PPC and during some targeted interventions by the MOMI project team and the district management responsible for reproductive health such as: upgrading the PPC skills of FHWs and solving issues related to high staff turnover and to the reluctant collaboration of some staff in HF dispensaries—where immunization usually was carried out—and in maternity wards.

HFs scheduled immunization services and infant checkups in clinics on the same weekday and some HFs even increased the number of planned days per week for vaccination in child clinics—usually from one to two days—in order to enhance the ability to integrate mother and infant PPC.

Another outcome of the project documents is that integrated maternal PPC utilization rate at week 6–8 with pentavalent dose 1 vaccine was insignificant. As shown in the previous section, the main raison was that the schedules for maternal PPC and immunization did not match. It seems that sensitization efforts of HF providers were not successful. Also, the staff seemed to be overloaded with infant immunization and visits and had no time to perform maternal PPC, which resulted in long waiting times for the mothers and discouraged them from attending maternal PPC.

Although the supervision confirms the utilization of checklists by health staff, the trend analysis shows that implementation of the supervision recommendations varied from one health facility (HF) to the other. Some recommendations were recurrent during supervision, for instance health information system registers filling. The decision to keep the mother health book in the HF until day 6–10 PP visit and the newborn BCG vaccine helped to increase day 6–10 PP visit utilization since mothers had to return to the facility for the visit and to get back the health book which is used for infant growing monitoring and immunization.

## Discussion

Our study findings show an improvement of PPC delivery for the mother–infant pair and of the integrated mother PPC during BCG vaccinations at day 6–10 PPC. The global project objective for the day 6–10 visit was reached. Counselling on FP was integrated into PP services and the indicator was close to the objective of 40%. PPC at week 6–8 remained weak and under the objective of 50% and the indicators suggest that it was not integrated into infant vaccination with pentavalent dose 1. PPC utilization increased more in rural facilities than in urban ones. Furthermore we noted a saturation in the urban advancement. Maternal and infant morbidity decreased during the implementation of the interventions. These findings are in line with those of the MOMI project end evaluation [9].

Several factors may explain the contrasting results between urban and rural facilities. First, the MOMI project end evaluation showed that the upstream work of female CHWs had a snowball effect in rural areas [9]. This type of community intervention was less efficient in urban areas because of difficulties in reaching the majority of the HF population living in urban slums [9, 20]. Second, integration seems to be more successful in rural facilities where HFs are usually staffed with a lower number of HW [21]. This fact, however, probably explains the relative success of the integration because collaboration and coordination efforts are easier to accomplish within small groups. Moreover, health service organizations are usually weak in urban areas, with leadership and management problems between maternity and dispensary provider, the latter being the manager of primary health care. Since both midwives and nurses usually have education levels equivalent to those of the managers, this situation created conflicts and lowered the motivation of health workers working in both units. These reasons induced difficulties of systematic integration in some health facilities. Third, the difference between the intervention implementation in rural and urban health facilities should be explained by the determinants of rapid urbanization in Kaya town. In the outskirts of the town, informal settlements or slums were being built, reflecting rapid urbanization which induced negative effects on health-related attitudes and behaviour, especially linked with the breaking of women’s social networks [22–24].

Key contextual factors of the successful upgrade of PPC interventions in Burkina Faso were the retention of the health book at the health facility until the day 6–10 visit and the female CHW intervention [9]. Sensitization through social networks with an appropriate timetable as implemented by the female CHW proved positive for behavioural change and raised attention to PPC [25, 26]. Monitoring and supervision were revealed to be an important factor of success for the interventions [9]. Dossa et al. in a systematic review found that Routine Health Information Systems as well as Intermittent Community Surveys should be increasingly used for impact studies on maternal and neonatal health in low- and middle-income countries [27]. On the other hand, our qualitative result somehow contradicted the result of a Cochrane review which found that the effect of supportive supervision on the quality of primary health care was uncertain [28, 29].

In our paper we used two databases, from the monitoring of indicators and from linked records between PPC and infant immunization, both using the routine health information system to explore the upgrade of PPC in 12 HFs where a package of interventions was implemented from September 2013 to December 2015. Results showed an increase in PP utilization rate although levels and trends varied depending on the health facility, and with large differences between urban and rural place of residence. Results from the linked mother PP and infant immunization data and from the documents review found a low integration of mother PP services in infant immunization particularly at the week 6–8 PP visit. The same outcome using direct observation and individual interviews was found during MOMI end evaluation [9].

Our paper detected a low quality in health system routine data. Indeed, the proportion of data on immunization and PPC visits which could be linked was very low. As mentioned, this could indicate the low success rate of the intervention but it is more likely to be due to the failure of the registration system, which is done manually. This deficiency has been shown in the context of a recent study carried out in 30 health facilities in Burkina Faso which found discrepancies in the immunization data, for instance between data in the immunization card, in the immunization registers and in the HF monthly reports [30]. Therefore upgrading and modernizing the registration system seems necessary in the context of Burkina Faso. Studies showed that the use of new technology seems acceptable and feasible in rural HFs and/or in resource-constrained settings, and the time needed for service providing in these studies does not increase [31, 32]. However, there are also challenges associated with the use of new technology that will not fix the quality issues of the health system [33].

Since 2005 and the launching of the Partnership for Maternal, Newborn and Child Health (PMNCH) consortium—which combined maternal, newborn and child mortality in a continuum of care and with other key health and development issues, such as HIV/AIDS and poverty reduction—the integration of MCH services has been advocated as the way forward to improve the providing of maternal health services [34–37]. Integrating MCH care allows to pool funding resources in a comparable manner as other pooled funding, such as the Global fund against Malaria, Tuberculosis and HIV/AIDS. It seems more efficient than the integration of service providing in practice [34]. In the specific case of PPC the factors and the challenge to ensure integration success need to be deeply analysed, looking at differences depending on the timing of PPC [38].

### Limitations

Outcomes on PPC attribution to MOMI projects should be discussed since it would require more thorough methods to exclude bias results for instance case control study and/or random sampling. Indeed, several MCH programmes and projects were implemented in the study site, e.g. Victory against Malnutrition (ViM), Performance-based financing (PBF)^3^ and the non-governmental organization Marie Stope International (MSI). The ViM project distributed food to mothers when they accomplished all health checks including week 6–8 PPC. PPC at day 6–8 and week 6 are indicators of PBF which has been piloted in Kaya district since March 2014. MSI was delivering FP services in the Kaya health district.

While it is worth noting that the sample of 12 primary HFs is neither representative of the 52 primary HFs within the Kaya health district nor of HFs overall in Burkina Faso, we think the data illustrate some of the issues and challenges faced by primary health care facilities in the country.

## Conclusion

Our study highlights the upgrade of PP integrated care at day 6–10 PP visit and of the management of postpartum morbidity as positive effects of the interventions. Strategies for the week 6–8 visit and for later visits need to be further developed, especially in relation to FP. Any upgrade of integration of RMNCH services needs to involve all actors, and to differentiate between rural and urban areas. Additional research is needed to evaluate the impact of such interventions and to explore the enablers and barriers as well as transferability of the results before deciding to scale it up.

## Additional files

Additional file 1: **Table S1.** Presents data by source and content. (DOCX 12 kb)

Additional file 2: **Figure S1.** Number of lives births per month per place of residence (rural, urban. Figure S1 shows the trend of live births over time in rural and urban HFs. Births follow a seasonal pattern linked to the Sudanese climate zone with dry and rainy seasons. Burkina Faso’s population is mainly rural (> 80%) and during the rainy season, from May to October, people are busy farming. Women become pregnant during the dry season, mainly from December to January, during which farming activities are less intense and temperatures are the lowest (17 °C). Therefore, live birth deliveries were more numerous in September 2013, 2014 and 2015. (DOCX 19 kb)

Additional file 3: **Table S2.** Monitored indicators by number of cases by health facility per period. Table S2 shows that there was an increase in the number of women who visited day 6–10 and week 6–8 PPC, and who received PPFP counselling and chose an FP method between September 2012 and August 2013 (1st period) and September 2015–December 2015 (2nd period), in both rural and urban areas. For instance in rural HFs, day 6–10 PPC increased from 751 visits in the 1st period to 2733 in the 2nd period, after two years of intervention. (DOCX 15 kb)

Additional file 4: **Table S3.** Comparison of PP visit utilization rate from the linked data and from the monitoring. Table S3 compares data from the two methods used (the matched records between the mother’s PP visit and the utilization of immunization services, and the monitoring data) during the same period, from September 2013 to August 2014. There is a difference at day 6–10 when PP visit utilization rate was generally lower with monitoring data compared to linked data, except in Delga, Kalambaogo and Tangasgo HFs. In general, comparability is better in rural HFs. Depending on the HF, the quality of the data differs according to the visit and probably according to the provider who fills in the registers. A more systematic registration of women and children through identification numbers could for instance improve the situation and facilitate the monitoring of activities. (DOCX 13 kb)

## Abbreviations

BCG: Bacillus Calmette-Guérin
CHW: Community health workers
DTPP: Diphtheria, tetanus, pertussis and poliomyelitis
FHWs: Facility health workers
FP: Family planning
HF: Health facility
HFs: Health facilities
HIV/AIDS: Human immunodeficiency virus infection and acquired immune deficiency syndrome
ITSA: Interrupted time series analysis
Kaya HDSS: Kaya demographic and health surveillance system
MCH: Maternal and child health services
MOMI: Missed opportunities for maternal and infant health
MSI: Marie Stope International
PBF: Performance-based financing
Penta + Hep1: DTP vaccine, haemophilus influenza type B, rotavirus and pneumococcal conjugate vaccine/dose 1 (pentavalent + Hep 1)
PHC: Primary health care
PP: Postpartum
PPC: Postpartum care
PPFP: Postpartum family planning
PPH: Postpartum hemorrhage
RMNCH: Reproductive, maternal, newborn and child health services
SDG: Sustainable development goal
ViM: Victory against malnutrition

## Footnotes

1 PPFP counselling was not chosen as an indicator as it matches exactly the day 6–10 PPC.

2 The regression model assumed the following form:

1 $$ {Y}_t={\beta}_0+{\beta}_1{T}_t+{\beta}_2{X}_t+{\beta}_3{X}_t{T}_t+{\epsilon}_t $$

We assume that the random error terms follow a first autoregressive process *ϵ*_*t*_ = *ϵ*_*t* − 1_ + *u*_*t*_. error terms, such that |ρ| < 1, and the disturbances *u*_*t*_ are independent N(0, *σ*^2^). *Y*_*t*_ represents the average number per month of day 6–10 PPC or week 8–10 PPC or PPFP choice, *T*_*t*_ is the time since the start of the study, *X*_*t*_ is a dummy (indicator) variable representing the intervention periods (pre-intervention periods 0, otherwise 1), and *X*_*t*_*T*_*t*_ is an interaction of *X*_*t*_ and *T*_*t*_. *β*_0_ represents the intercept, or starting level of the outcome variable, *β*_1_ the slope, or trajectory of the outcome variable until the introduction of the intervention, and *β*_2_ the change in the level of the outcome that occurs in the period immediately following the introduction of the intervention (compared to the counterfactual). *β*_3_ is the difference between pre- and post-intervention slopes of the outcome. Thus we look for significant *p*-values in *β*_2_ to indicate an immediate treatment effect, or in *β*_3_ to indicate a treatment effect over time. Finally, *β*_1_ + *β*_3_ is the post-intervention trend.

3 Performance based financing is a “strategy for using explicit performance-based subsidies to support the delivery of basic services where policy concerns would justify public funding to complement or replace user-fees. The core of the approach is the contracting out of service delivery to a third party, where payment of public funds is tied to the actual delivery of these services”. (p. 1)
