# Supplementary material for: Integration of maternal postpartum services in maternal and child health services in Kaya health district (Burkina Faso): an intervention time trend analysis
Source: BMC Health Serv Res. 2018 Apr 23;18:298. doi: 10.1186/s12913-018-3098-6 (PMC5914017; doi:10.1186/s12913-018-3098-6)
Supplement: Supplementary file 1 — Table S1. Presents data by source and content. (DOCX 12 kb) [file 12913_2018_3098_MOESM1_ESM.docx]

**Additional file1**

**Table S1: Data by source and content**

| **Data** | **Source** | **Details** | **Period** |
| --- | --- | --- | --- |
| Quantitative study | | | |
| Monitoring of indicators | Postpartum registers | Up to 40 monthly observations of indicators | From September 2012 to December 2015 |
| Linked data from postpartum and immunization registers | Postpartum registers  Immunization registers | Date of visits  Name of infant, Name of mother, Health facility | From September 2013 to August 2014 |
| Qualitative study | | | |
| Quarterly supervision reports | Project reports | 9 supervision reports | From October 2013 to December 2015 |
| Log frame | Project documents |  |  |
| Implementation plan, Checklist, protocols, Baseline study report, | Project documents |  | From February 2012 to March 2016 |
