# Supplementary material for: Integration of maternal postpartum services in maternal and child health services in Kaya health district (Burkina Faso): an intervention time trend analysis
Source: BMC Health Serv Res. 2018 Apr 23;18:298. doi: 10.1186/s12913-018-3098-6 (PMC5914017; doi:10.1186/s12913-018-3098-6)
Supplement: Supplementary file 2 — Figure S1. Number of lives births per month per place of residence (rural, urban. Figure S1 shows the trend of live births over time in rural and urban HFs. Births follow a seasonal pattern linked to the Sudanese climate zone with dry and rainy seasons. Burkina Faso’s population is mainly rural (> 80%) and during the rainy season, from May to October, people are busy farming. Women become pregnant during the dry season, mainly from December to January, during which farming activities are less intense and temperatures are the lowest (17 °C). Therefore, live birth deliveries were more numerous in September 2013, 2014 and 2015. (DOCX 19 kb) [file 12913_2018_3098_MOESM2_ESM.docx]

**Figure S1: Number of live births per month per place of residence (rural, urban)**

**Additional file2**
