# Supplementary material for: Integration of maternal postpartum services in maternal and child health services in Kaya health district (Burkina Faso): an intervention time trend analysis
Source: BMC Health Serv Res. 2018 Apr 23;18:298. doi: 10.1186/s12913-018-3098-6 (PMC5914017; doi:10.1186/s12913-018-3098-6)
Supplement: Supplementary file 3 — Table S2. Monitored indicators by number of cases by health facility per period. Table S2 shows that there was an increase in the number of women who visited day 6–10 and week 6–8 PPC, and who received PPFP counselling and chose an FP method between September 2012 and August 2013 (1st period) and September 2015–December 2015 (2nd period), in both rural and urban areas. For instance in rural HFs, day 6–10 PPC increased from 751 visits in the 1st period to 2733 in the 2nd period, after two years of intervention. (DOCX 15 kb) [file 12913_2018_3098_MOESM3_ESM.docx]

**Additional file 3**

**Table S2: Monitored indicators by number of cases by health facility per period**

| **Primary Health Facilities** | | **Number of live-birth deliveries** | | | | **Number of pair mother newborn/infant who received postpartum care at day 6-10** | | | | **Number of pair mother newborn/infant who received postpartum care at week 6-8** | | | | **Number of women who used a PPFP method** | | | |
| --- | --- | --- | --- | --- | --- | --- | --- | --- | --- | --- | --- | --- | --- | --- | --- | --- | --- |
|  |  | Sept12-Aug13 | Sept13-Aug14 | Sept14-Aug15 | Sept15-Dec15 | Sept12-Aug13 | Sept13-Aug14 | Sept14-Aug15 | Sept15-Dec15 | Sept12-Aug13 | Sept13-Aug14 | Sept14-Aug15 | Sept15-Dec15 | Sept12-Aug13 | Sept13-Aug14 | Sept14-Aug15 | Sept15-Dec15 |
| **Rural HF** | **Basnere** | 365 | 383 | 446 | 132 | 158 | 381 | 439 | 132 | 57 | 232 | 347 | 96 | 8 | 10 | 130 | 38 |
|  | **Damesma** | 198 | 236 | 202 | 65 | 31 | 197 | 188 | 50 | 9 | 83 | 92 | 5 | 0 | 86 | 85 | 3 |
|  | **Delga** | 237 | 320 | 273 | 120 | 23 | 295 | 257 | 68 | 25 | 202 | 233 | 52 | 0 | 43 | 71 | 9 |
|  | **Kalambaogo** | 317 | 407 | 431 | 123 | 0 | 291 | 364 | 129 | 0 | 83 | 103 | 54 | 0 | 55 | 277 | 61 |
|  | **Lebda** | 243 | 331 | 277 | 102 | 293 | 320 | 363 | 113 | 129 | 134 | 120 | 41 | 0 | 45 | 153 | 22 |
|  | **Namsigui** | 349 | 374 | 422 | 144 | 64 | 261 | 376 | 129 | 4 | 66 | 171 | 79 | 0 | 68 | 182 | 12 |
|  | **Napalgue** | 265 | 303 | 292 | 102 | 25 | 264 | 357 | 139 | 3 | 67 | 90 | 48 | 1 | 41 | 217 | 86 |
|  | **Tangasco** | 166 | 136 | 146 | 53 | 157 | 373 | 389 | 127 | 167 | 301 | 349 | 107 | 60 | 46 | 81 | 9 |
|  | ***Total rural HF*** | ***2140*** | ***2490*** | ***2489*** | ***841*** | ***751*** | ***2382*** | ***2733*** | ***887*** | ***394*** | ***1168*** | ***1505*** | ***482*** | ***69*** | ***394*** | ***1196*** | ***240*** |
| **Urban HF** | **Sector 1** | 699 | 752 | 798 | 263 | 176 | 246 | 320 | 100 | 144 | 145 | 261 | 92 | 0 | 18 | 83 | 38 |
|  | **Sector 4** | 372 | 429 | 454 | 166 | 0 | 189 | 347 | 121 | 0 | 65 | 304 | 112 | 0 | 26 | 40 | 71 |
|  | **Sector 6** | 417 | 611 | 603 | 206 | 46 | 213 | 266 | 95 | 31 | 97 | 252 | 82 | 0 | 26 | 78 | 24 |
|  | **Sector 7** | 416 | 424 | 534 | 199 | 41 | 130 | 161 | 48 | 9 | 73 | 120 | 58 | 0 | 11 | 59 | 49 |
|  | ***Total urban HF*** | ***1904*** | ***2216*** | ***2389*** | ***834*** | ***263*** | ***778*** | ***1094*** | ***364*** | ***184*** | ***380*** | ***937*** | ***344*** | ***0*** | ***81*** | ***260*** | ***182*** |
| ***Total HF*** | | ***4044*** | ***4706*** | ***4878*** | ***1675*** | ***1014*** | ***3160*** | ***3827*** | ***1251*** | ***578*** | ***1548*** | ***2442*** | ***826*** | ***69*** | ***475*** | ***1456*** | ***422*** |
