# Supplementary material for: Integration of maternal postpartum services in maternal and child health services in Kaya health district (Burkina Faso): an intervention time trend analysis
Source: BMC Health Serv Res. 2018 Apr 23;18:298. doi: 10.1186/s12913-018-3098-6 (PMC5914017; doi:10.1186/s12913-018-3098-6)
Supplement: Supplementary file 4 — Table S3. Comparison of PP visit utilization rate from the linked data and from the monitoring. Table S3 compares data from the two methods used (the matched records between the mother’s PP visit and the utilization of immunization services, and the monitoring data) during the same period, from September 2013 to August 2014. There is a difference at day 6–10 when PP visit utilization rate was generally lower with monitoring data compared to linked data, except in Delga, Kalambaogo and Tangasgo HFs. In general, comparability is better in rural HFs. Depending on the HF, the quality of the data differs according to the visit and probably according to the provider who fills in the registers. A more systematic registration of women and children through identification numbers could for instance improve the situation and facilitate the monitoring of activities. (DOCX 13 kb) [file 12913_2018_3098_MOESM4_ESM.docx]

**Additional file 4**

**Table S3: Comparison of PP visit utilization rate from the linked data and from the monitoring**

| **Primary health facility** | | **Day 6-10 PP visit** | |
| --- | --- | --- | --- |
|  |  | % from linked data | % from monitoring September 2013-August 2014 |
| **Rural HF** | **Basnere** | 88% | 99% |
|  | **Damesma** | 95% | 83% |
|  | **Delga** | 84% | 92% |
|  | **Kalambaogo** | 80% | 92% |
|  | **Lebda** | 95% | 74% |
|  | **Namsigui** | 86% | 51% |
|  | **Napalgue** | 91% | 70% |
|  | **Tangasgo** | 85% | 96% |
|  | ***Total rural HF*** | ***88%*** | ***81%*** |
| **Urban HF** | **Sector 1** | 89% | 39% |
|  | **Sector 4** | 83% | 75% |
|  | **Sector 6** | 82% | 43% |
|  | **Sector 7** | 89% | 62% |
|  | ***Total urban HF*** | ***86%*** | ***51%*** |
| ***Total*** | | ***87%*** | ***67%*** |
